# GADD45B Transcript Is a Prognostic Marker in Papillary Thyroid Carcinoma Patients Treated With Total Thyroidectomy and Radioiodine Therapy

**DOI:** 10.3389/fendo.2020.00269

**Published:** 2020-04-30

**Authors:** Mateus C. Barros-Filho, Julia B. H. de Mello, Fabio A. Marchi, Clóvis A. L. Pinto, Igor C. da Silva, Patricia K. F. Damasceno, Milena B. P. Soares, Luiz P. Kowalski, Silvia R. Rogatto

**Affiliations:** ^1^International Research Center–CIPE, A. C. Camargo Cancer Center, São Paulo, Brazil; ^2^Department of Pathology, A. C. Camargo Cancer Center, São Paulo, Brazil; ^3^Department of Pathology, São Rafael Hospital, Salvador, Brazil; ^4^Gonçalo Moniz Institute, Fiocruz, Salvador, Brazil; ^5^Health Technology Institute, SENAI CIMATEC, Salvador, Brazil; ^6^Department of Head and Neck Surgery and Otorhinolaryngology, A. C. Camargo Cancer Center, São Paulo, Brazil; ^7^Department of Clinical Genetics, Vejle University Hospital, Institute of Regional Health Research, University of Southern Denmark, Odense, Denmark

**Keywords:** papillary thyroid cancer, prognostic markers, BRAF mutation, *GADD45B*, TERT promoter mutation, transcription profiling

## Abstract

Currently, there is a lack of efficient recurrence prediction methods for papillary thyroid carcinoma (PTC). In this study, we enrolled 202 PTC patients submitted to total thyroidectomy and radioiodine therapy with long-term follow-up (median = 10.7 years). The patients were classified as having favorable clinical outcome (PTC-FCO, no disease in the follow-up) or recurrence (PTC-RE). Alterations in *BRAF, RAS, RET*, and *TERT* were investigated (*n* = 202) and the transcriptome of 48 PTC (>10 years of follow-up) samples was profiled. Although no mutation was associated with the recurrence risk, 68 genes were found as differentially expressed in PTC-RE compared to PTC-FCO. Pathway analysis highlighted a potential role of cancer-related pathways, including signal transduction and FoxO signaling. Among the eight selected genes evaluated by RT-qPCR, *SLC2A4* and *GADD45B* showed down-expression exclusively in the PTC-FCO group compared to non-neoplastic tissues (NT). Increased expression of *GADD45B* was an independent marker of shorter disease-free survival [hazard ratio (HR) 2.9; 95% confidence interval (CI95) 1.2–7.0] in our cohort and with overall survival in the TCGA dataset (HR = 4.38, CI95 1.2–15.5). In conclusion, *GADD45B* transcript was identified as a novel prognostic marker candidate in PTC patients treated with total thyroidectomy and radioiodine therapy.

## Introduction

The incidence of thyroid carcinoma has tripled over the last 35 years, affecting more than 560,000 people worldwide in 2018 (1). Papillary thyroid carcinoma (PTC) represents 80–85% of all thyroid cancer, presenting a high cure rate and a 5-years overall survival of 98% (2). However, recurrence is a frequent event (10–25%) related to patient morbidity and may occur over 20 years after the initial treatment (3, 4). Clinical-pathological features, such as distant and lymph node metastasis, extrathyroidal extension, and tall cell histologic variant, are associated with more aggressive PTC (5). Nonetheless, the discovery of reliable biomarkers to determine the risk of relapse could be of great value in clinical practice. Low-risk PTC may be eligible for minimalistic surgical approaches (such as thyroid lobectomy) or active surveillance. On the other hand, a more aggressive intervention (such as total thyroidectomy, prophylactic neck dissection, or radioiodine therapy) could be reserved for high risk PTC (5).

The most common genetic driver alterations found in PTC are *BRAF* (60%) and *RAS* (13%) point mutations, *TERT* promoter mutation (9%), and *RET/PTC* fusion (6%) (6). *BRAF* and *TERT* mutations have been frequently associated with more aggressive thyroid carcinomas (7–10). Although the coexistence of both alterations has a synergic effect (11), their role in the prognosis of PTC is still controversial (12, 13).

Gene expression profiling has been widely evaluated in thyroid cancer for biomarker discovery, especially for diagnostic purposes (14–17). Transcriptomic-based studies have revealed predictors candidates of prognosis, including overexpression of *MUC1* (18), *MEDAG* (19), and *SPHK1* (20), and down-expression of *FMO1* (21), and *FOXF1* (22). Signatures of multigene classifiers were also reported (23, 24). Although many prognostic candidates have been suggested, most of them were not confirmed in distinct cohorts (25). The inclusion of a limited number of patients treated with or without radioiodine and followed by short periods could explain the lack of reproducibility.

In this study, we evaluated a cohort of 202 PTC patients (69% of them with more than 10 years of follow-up) with standardized treatment (total thyroidectomy and radioiodine therapy). We reported that the most common genomic alterations (*BRAF, RAS, RET*, and *TERT*) found in PTC were not related to the recurrence risk in the long-term follow-up. The transcriptomic profiling (microarray) revealed potential recurrence biomarkers, of which a higher expression of *GADD45B* (Growth arrest and DNA-damage-inducible, beta) was an independent marker of shorter disease-free survival (confirmed by RT-qPCR).

## Materials and Methods

### Patient Selection Criteria

Patients with pathological confirmation of PTC treated from July 2001 to December 2010 at A. C. Camargo Cancer Center, São Paulo, Brazil, were retrospectively included in this study. The samples were selected according to the availability of fresh-frozen tissues at our BioBank. The Ethics Committee in Human Research for the Institution approved this study (Protocol n° 1410/10), which was conducted according to the Helsinki Declaration. The tumor specimens obtained from the thyroidectomy were reviewed by an experienced pathologist (CP) using blinded interpretation.

In order to standardize the treatment strategy used in our cohort, only patients submitted to total thyroidectomy followed by radioiodine therapy were enrolled. Patients with other cancer types prior to the thyroid cancer diagnosis were excluded to avoid bias in the prognostic analysis. We also excluded samples with low RNA quality (RNA integrative number < 5). Patients with no evidence of active disease at the follow-up, defined as negative image test by ultrasonography and serum thyroglobulin (< 1 ng/mL with suppressed TSH), were classified as having favorable clinical outcome (FCO). Recurrence (RE) was defined as persistent or recurrent PTC after the definitive treatment with pathologic confirmation (fine-needle aspiration biopsy or surgery) or combined imaging (Computed Tomography or Positron Emission Tomography with Computed Tomography) and strong biochemical evidence (persistent serum thyroglobulin> 2 ng/ml with suppressed TSH< 0.1 mIU/L or thyroglobulin> 5 ng/ml with induced TSH> 30 mIU/L). Due to the presence of late recurrence during the natural history of PTC (3), we have included only patients followed up for more than 5 years in the FCO group. Based on the fact that the microarray assays were used as a “discovery set,” we adopted a minimum follow-up of 10 years.

Following these criteria, a total of 202 patients were included (Table S1). The sample distribution according to the molecular approaches is summarized in Figure S1. We also included 15 non-neoplastic thyroid (NT) tissues in the RT-qPCR analysis. The NT tissues were obtained from surrounding PTC samples showing no histological alterations, hyperplastic, or inflammatory changes in the remaining thyroid parenchyma.

### Detection of Genomic Alterations

Nucleic acids (DNA and RNA) were isolated, as previously described (26). Adequate quantity and quality for 202 DNA and 178 RNA PTC specimens were obtained. Point mutations in *BRAF* (codon 600), *KRAS* (codon 12/13), *HRAS* (codon 61), and *KRAS* (codon 61) were evaluated by pyrosequencing and *RET* rearrangements (*RET*/PTC1 and *RET*/PTC3) by RT-qPCR, as previously described (27). *TERT* promoter mutations (C228T and C250T hotspots) were investigated by direct Sanger sequencing, as described elsewhere (28).

### Gene Expression Profiling

Gene expression microarray experiments were performed in 48 PTC using the SurePrint G3 8x60K platform (Agilent Technologies Inc., Santa Clara, CA, USA), co-hybridized with a pool of nine non-neoplastic thyroid tissues, as previously described (15). This data was generated in a previous study (15) and is available in the GEO database (accession number GSE50901). The probes representing protein-coding genes were selected and quantile-normalized using BRB ArrayTools software (v. 4.4.0). Groups were compared using the limma package (*P* < 0.01) (29), adopting a fold change (FC) ≥ 1.5 to define differential expression. Since male patients usually present a worse prognosis (30), representing a potential bias in our study, genes found as more or less expressed according to gender and mapped in *X* or *Y* chromosomes were excluded. Hierarchical clustering analysis was performed with Euclidean distance and complete linkage using ComplexHeatmap package (31) available for R program.

### *In silico* Molecular Analysis

Genes differentially expressed identified in the microarray analysis were subjected to an *in silico* exploration, employing two pathway-enrichment tools, KOBAS (v.3.0; kobas.cbi.pku.edu.cn/) and pathDIP (http://ophid.utoronto.ca/pathdip/), using KEGG, Reactome and PANTHER databases. Experimentally detected and computational predicted protein-protein interactions (minimum confidence level for predicted associations of 99%) were used in the pathDIP tool, while literature curated known pathway memberships were used in KOBAS. Pathways highlighted by both tools were designated as putatively disrupted (hypergeometric test with Benjamini and Hochberg correction *P* < 0.05).

### Reverse Transcription Quantitative PCR (RT-qPCR) Analysis

Eight genes (*ELMO1, F2RL2, FOXP2, GADD45B, HGD, JUND, S1PR1*, and *SLC2A4*) were selected for RT-qPCR investigation using TaqMan Low Density Arrays® (TLDA; Applied Biosystems, Foster City, CA, USA) in 72 PTC, including 38 samples tested prior by microarray, 34 independent cases, and 15 additional non-neoplastic thyroid samples (histological normal pattern tissue surrounding tumor). The gene selection considered the *P*-value (*FOXP2, GADD45B, HGD, JUND*, and *SLC2A4* were among the top 15 lowest *P*-values), fold change (*F2RL2* had the highest FC) and pathway analysis (*ELMO, GADD45B, S1PR1*, and *SLC2A4* were members of FoxO signaling or Signal Transduction pathways). Two references (*EIF2B1* and *PUM1*) were selected among five transcripts (*18S, EIF2B1, PUM1, TBP*, and *YWHAZ*) using geNorm (32) to obtain the normalized target gene relative expression. *GADD45B* (target) and reference genes (*EIF2B1* and *PUM1*) were further evaluated in 106 PTCs using individual Taqman assays (Applied Biosystems, Hs04188837_g1, Hs00426752_m1 and Hs00472881_m1, respectively). The reactions were assembled in duplicates (10 ng of cDNA) according to the manufacturer instructions, using automatic pipetting (QIAgility, QIAGEN, Courtaboeuf, France). The amplifications were carried out with 7900HT Real Time PCR System (Applied Biosystems). Normalization was implemented following the Pfaffl method (33).

### TCGA Database

Disease-free survival, overall survival, *GADD45B* expression (RNA sequencing, log_2_ transformed RSEM+1), and *BRAF* mutation (exome sequencing) data from PTC patients were retrieved from the UCSC Xena Browser (https://xenabrowser.net/datapages/, accessed in October 2019). In total, 490 PTC subjects had both follow-up and gene expression information available for the analysis.

### Statistical Analysis

Statistical analysis and illustrations were performed with BRB ArrayTools (v. 4.4.0), SPSS (v. 21.0; SPSS, Chicago, IL, USA) and Graphpad Prism (v. 5.0; GraphPad Software Inc., La Jolla, CA, USA) software. Genomic alterations were confronted with clinical-pathological features using Fisher exact test with multiple hypothesis correction (Bonferroni test). Relative expression obtained by RT-qPCR was compared among biological groups with Student *t*-test and ANOVA (Tukey *post-hoc* test). A two-tailed *P* < 0.05 value was adopted as significant. Gene expression values were dichotomized in bellow and above the median (RT-qPCR from our cohort and RNA sequencing from TCGA) to perform the survival analyses. The Kaplan–Meier method was used to plot the disease-free and overall survival. Cox proportional-hazards regression was used in the univariate and multivariate survival analysis to estimate the hazard ratio (HR) and 95% confidence intervals (CI95). Variables significantly associated (*P* < 0.05) in the univariate were included in the multivariate model (conditional backward elimination) (SPSS (v. 21.0; SPSS, Chicago, IL, USA).

## Results

### PTC Relapse Risk Was Not Associated With *BRAF, RAS, RET*, and *TERT* Alterations

We detected *BRAF*V600E mutation in 62.4% (126/202) of our cases. *RAS* mutation was found in 2.5% (5/198) (all in *NRAS*), *TERT* promoter mutations in 2.6% (5/193; 1 C228T and 4 C250T), and *RET* rearrangement in 9.2% (16/174; 11 *RET/PTC1* and 5 *RET/PTC3*) of the tumors. Only two cases presented concurrent alterations in *BRAF, RAS*, and *RET*, one classical variant (*BRAF* and *RET/PTC*), and one follicular variant (*BRAF* with *NRAS*). Four of five *TERT* positive cases also presented *BRAF* mutations. These four *BRAF/TERT* concurrent mutations were from patients older than 55 years, tumors larger than 1 cm with extrathyroidal extension. Three were classic variants, and the patients had a favorable clinical outcome. One patient presented a diffuse sclerosing variant of PTC that progressed with distant metastasis and died due to the disease. Tumors harboring *BRAF* mutations were correlated with the classical variant of PTC (*P* = 0.007) and the presence of extra-thyroidal extension (*P* = 0.025). However, no significant association was found after a multiple-comparison correction (Table 1). *RAS* mutations were prevalent in the follicular variant, *TERT* in older patients, and *RET/PTC* in patients with lymph node metastasis (all significant after the multiple-comparison correction). No significant difference was observed between the assessed alterations with the risk of relapse (Table 1).

**Table 1:** Clinical-pathological characteristics according to the status of *BRAF, RAS*, and *TERT* mutations and *RET* rearrangements in papillary thyroid carcinomas.

**Variables**	***BRAF* (*N* = 202)**		***RAS* (*N* = 198)**		***TERT* (*N* = 193)**		***RET* (*N* = 174)**	
	**Mutated/total (%)**	***P***	**Mutated/total (%)**	***P***	**Mutated/total (%)**	***P***	**Fusion/total (%)**	***P***
**Age**								
<55 years	106/175 (61)	0.206	5/171 (3)	1.000	1/167 (1)	**0.001**	16/150 (11)	0.132
≥55 years	20/27 (74)		0/27 (0)		4/26 (15)		0/24 (0)	
**Gender**								
Female	95/155 (61)	0.609	3/152 (2)	0.330	4/149 (3)	1.000	11/135 (8)	0.359
Male	31/47 (66)		2/46 (4)		1/44 (2)		5/39 (13)	
**Tumor Size**								
≤1 cm	58/91 (64)	0.771	2/89 (2)	1.000	0/85 (0)	0.068	6/78 (8)	0.606
>1 cm	68/111 (61)		3/109 (3)		5/108 (5)		10/96 (10)	
**Multifocal**								
No	67/113 (59)	0.464	3/112 (3)	1.000	3/107 (3)	1.000	10/97 (10)	0.793
Yes	55/85 (65)		2/82 (2)		2/82 (2)		6/73 (8)	
**Variant**								
Classical	103/150 (69)	0.007	0/147 (0)	**<0.001**	3/142 (2)	0.571	12/132 (9)	0.090
Follicular	16/37 (43)		4/36 (11)		1/36 (3)		1/30 (3)	
Other^a^	7/15 (47)		1/15 (7)		1/15 (7)		3/12 (25)	
**Invasion^b^**								
No	112/179 (63)	0.804	5/175 (3)	1.000	3/170 (2)	0.080	12/156 (8)	0.130
Yes	11/19 (58)		0/19 (0)		2/19 (11)		3/15 (20)	
**ETE**								
No	64/116 (55)	0.025	5/112 (4)	0.077	1/113 (1)	0.081	9/101 (9)	0.795
Yes	57/80 (71)		0/80 (0)		4/74 (5)		7/69 (10)	
**Node Status**								
cN0, pN0	83/134 (62)	0.879	5/133 (4)	0.174	4/127 (3)	0.662	5/119 (4)	**0.002**
pN1	43/68 (63)		0/65 (0)		1/66 (2)		11/55 (20)	
**Recurrence^c^**								
No	105/169 (62)	1.000	5/167 (3)	1.000	4/161 (2)	1.000	14/152 (9)	1.000
Yes	21/33 (64)		0/31 (0)		1/32 (3)		2/22 (9)	

*N, number of samples tested; ETE, extrathyroidal extension; P-value, Fisher exact test; in bold, statistically significant after Bonferroni correction (P = 0.05 divided by nine variables = P < 0.0056)*.

a *tall cells, solid, oncocytic, sclerosing and mucosecretory histological variants*.

b *Vascular and/or perineural invasions*.

c *Locoregional recurrence (N = 29) and distant metastases (N = 4, all in the lung)*.

### Gene Expression Profile as a Predictor of PTC Recurrence

**Figure 1:**
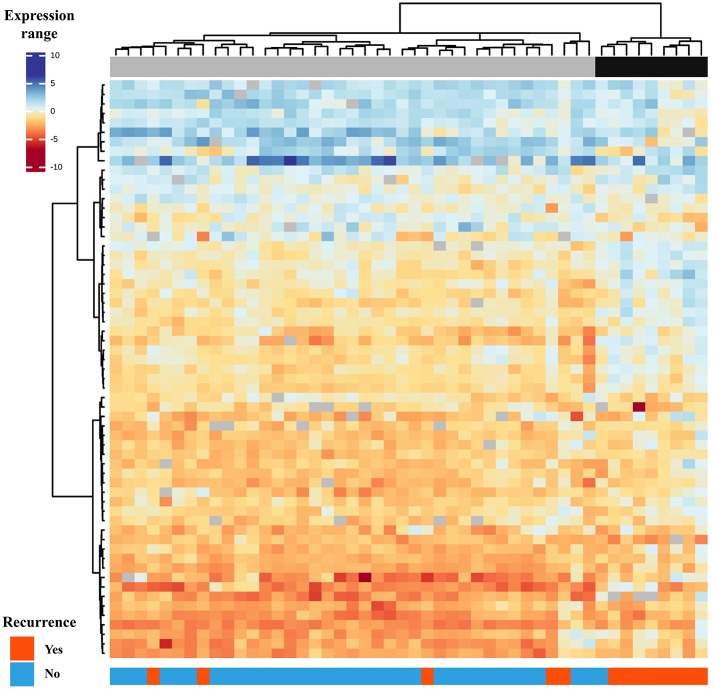
Supervised hierarchical clustering analysis comprising 61 genes differentially expressed in primary PTC-RE compared to PTC-FCO (samples in columns and genes in rows). Two major groups are shown: the first (gray) is enriched by patients with favorable clinical outcomes and the second (black) by patients who relapsed in the follow-up.

Gene expression profile of PTC from patients with recurrence (PTC-RE) was compared with PTC from patients with favorable clinical outcome (PTC-FCO). The microarray analysis unveiled 61 differentially expressed genes (17 less and 44 more expressed in PTC-RE compared to PTC-FCO) (Table S2). A supervised hierarchical clustering analysis including the differentially expressed genes revealed a “high risk” group comprising eight of 13 PTC-RE, and a “low risk” group containing 34 of 35 PTC-FCO (Figure 1).

### Potentially Disrupted Pathways Associated With Recurrence in PTC

To better understand the gene list obtained in the microarray analysis, we performed an *in silico* molecular analysis using pathDIP (http://ophid.utoronto.ca/pathdip/) and KOBAS (v.3.0; kobas.cbi.pku.edu.cn/) tools. We found an enrichment of signal transduction, peptide ligand-binding receptors, FoxO signaling, and platelet activation, signaling and aggregation pathways (*P* adjusted < 0.05) (Table 2).

**Table 2:** Biological pathways potentially altered in PTC from relapsed patients using KOBAS 3.0 and PathDip tools.

**Pathway name**	**Deregulated genes in pathway**	**KOBAS 3.0**		**PathDIP**	
		***P***	***FDR***	***P***	***FDR***
Signal transduction (Reactome)	*F2RL3, FLRT1, APOA1, ELMO1, PPP1R15A, CHEK1, GNA14, F2RL2, RAG2, CCL25, JUNB, GPR83, RHOB, S1PR1*	<0.001	0.001	<0.001	0.024
Platelet activation, signaling and aggregation (Reactome)	*PCDH7, APOA1, GNA14, F2RL2, RHOB, F2RL3*	<0.001	0.001	<0.001	0.019
FoxO signaling pathway (KEGG)	*S1PR1, SLC2A4, GADD45B, RAG2*	<0.001	0.003	<0.001	0.023
Peptide ligand-binding receptors (Reactome)	*CCL25, F2RL2, F2RL3*	0.004	0.049	<0.001	0.023

*P-value from hypergeometric test; False Discovery Rate (FDR) estimated by Benjamini-Hochberg method*.

### Confirmation of Genes Differentially Expressed in PTC by RT-qPCR and Their Association With Clinical Outcome

**Figure 2:**
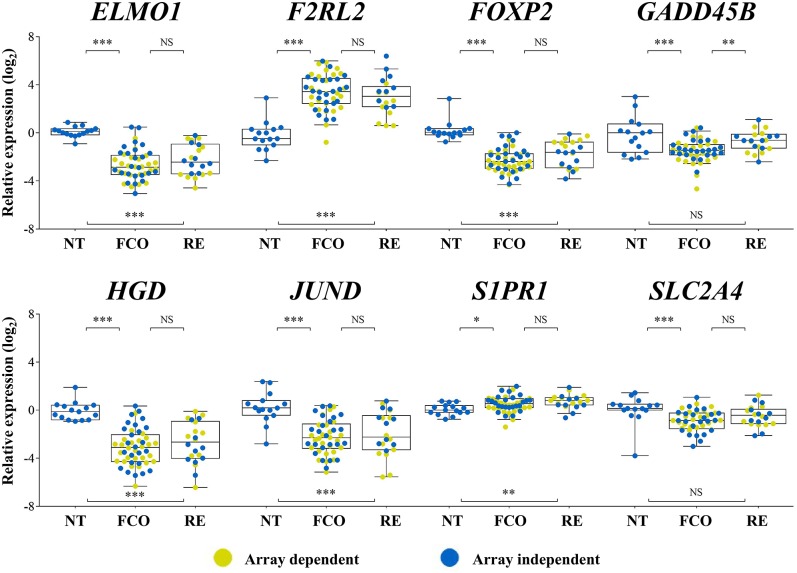
Expression levels of eight genes evaluated by RT-qPCR (TLDA custom assay) in PTC samples previously investigated by microarray analysis (yellow dots) and in an array-independent set of samples (blue dots). *GADD45B* showed statistically significant higher expression levels in PTC-RE compared to PTC-FCO cases. FCO, favorable clinical outcome; RE, recurrence; ***P < 0.001; **P < 0.01; *P < 0.05; NS, not significant (Tukey *post-hoc* test).

Eight targets (*ELMO1, F2RL2, FOXP2, GADD45B, HGD, JUND, S1PR1*, and *SLC2A4*) and two reference genes (*EIF2B1* and *PUM1*) were assayed by RT-qPCR (*N* = 72; TLDA method). *ELMO1, FOXP2, HGD*, and *JUND* were less expressed, and *F2RL2* and *S1PR1* more expressed in both PTCs groups compared to non-neoplastic thyroid tissues. *GADD45B* and *SLC2A4* were less expressed only in the PTC-FCO group compared to NT. However, only *GADD45B* showed a significant difference between the PTC-RE and PTC-FCO (more expressed in PTC-RE) (Figure 2).

### GADD45B Expression as a Prognostic Marker

**Figure 3:**
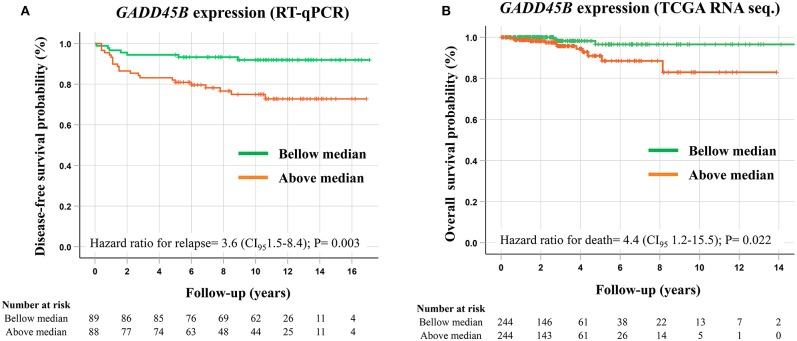
Survival analysis performed according to the *GADD45B* expression levels. **(A)** Kaplan–Meier plot demonstrating shorter disease-free survival in patients presenting higher *GADD45B* expression levels (above median) evaluated by RT-qPCR. **(B)** Kaplan–Meier plot showing a shorter overall survival in cases presenting higher *GADD45B* expression in PTC patients from the TCGA database (RNA sequencing). *P*-values were obtained by Cox proportional-hazards regression, and median expression was used as a cut-off.

A total of 106 PTC samples with available RNA was used to evaluate the *GADD45B* expression level by RT-qPCR assays using *EIF2B1* and *PUM1* as references. Combining both PCR sets (TLDA and Taqman individual assay), a cohort with 178 PTC samples was established. A higher expression of *GADD45B* (median expression as the threshold) was confirmed as a factor related to shorter disease-free survival (HR = 3.6, CI95 1.5–8.4; *P* = 0.003) (Figure 3A). Multivariate analysis revealed that *GADD45B* and cervical lymph node metastasis are independent predictors markers of relapse (*P* = 0.015, HR = 2.9, and *P* = 0.009, *HR* = 3.0, respectively) (Table 3).

**Table 3:** Univariate and multivariate analysis, contrasting the risk of relapse of PTC patients with clinical, pathological and molecular features.

**Variables**	**Univariate analysis**		**Multivariate analysis**	
	**HR (CI_95%_)**	***P***	**HR (CI_95%_)**	***P***
**Age**				
<55 years	1.0			
≥55 years	0.6 (0.2–2.1)	0.476		
**Gender**				
Female	1.0		1.0	
Male	2.3 (1.1–4.6)	**0.020**	1.6 (0.7–3.4)	0.241
**Tumor Dimension**				
≤1 cm	1.0			
>1 cm	2.0 (1.0–5.0)	0.062		
**Multicentricity**				
No	1.0			
Yes	2.0 (1.0–4.0)	0.062		
**Histological Variant**				
Classical	1.0			
Other	2.0 (0.8–5.2)	0.148		
**Vascular/Perineural Invasion**				
No	1.0		1.0	
Yes	3.2 (1.4–7.4)	**0.007**	1.8 (0.6–4.9)	0.271
**Extrathyroidal Extension**				
No	1.0			
Yes	1.8 (0.9–3.7)	0.091		
**Lymph Node Metastasis**				
No	1.0		1.0	
Yes	4.1 (2.0–8.4)	**<0.001**	3.0 (1.3–6.9)	**0.009**
***BRAF* Mutation**				
No	1.0			
Yes	1.1 (0.5–2.2)	0.867		
***RAS* Mutation**				
No	1.0			
Yes	0 (0–848.7)	0.542		
***RET/PTC***				
No	1.0			
Yes	1.0 (0.2–4.4)	0.972		
***TERT* Promoter Mutation**				
No	1.0			
Yes	1.4 (0.2–10.4)	0.728		
***GADD45B* Expression**				
Bellow median	1.0		1.0	
Above median	3.6 (1.5–8.4)	**0.003**	2.9 (1.2–7.0)	**0.015**

*HR, Hazard ratio; CI, confidence interval. P-value, Cox proportional-hazards regression. The bold values are statistically significant*.

Using the RNA sequencing data of PTC (*N* = 490) from the TCGA database (no standardized treatment), *GADD45B* also exhibited a prognostic role, being more expressed in patients with shorter overall survival (overall survival analysis: *HR* = 4.38, CI95 1.2–15.5; *P* = 0.022) (Figure 3B). However, no association with the recurrence risk was observed (disease-free survival analysis: *HR* = 0.69, CI95 0.35–1.4; *P* = 0.279) (Figure S2).

## Discussion

In general, PTC is an indolent disease and recurrence can appear long periods after surgery, making the identification of molecular prognostic markers a challenge (34). Herein, we investigated the most common gene alterations described in PTC, as well as transcriptomic data, to identify markers able to anticipate the outcome of patients treated with total thyroidectomy followed by radioiodine therapy. Only patients with a minimum follow-up of 10 years were included in the large-scale gene expression analysis, while specific mutations/rearrangements and mRNAs levels were evaluated in patients followed for at least 5 years.

Among the 202 PTC cases evaluated in this study, *BRAF* mutation was detected with a high frequency (62.4%), while *RAS* mutation (2.5%), *RET* fusions (9.2%), and *TERT* promoter mutation (2.6%) were uncommon. These frequencies are comparable to the ones available in the TCGA database (*BRAF*: 59.7%, *RAS*: 13% *RET*: 6.3% *TERT*: 9.4%) (6). Although reports in the literature show that *BRAF* and *TERT* mutations are related to aggressive thyroid tumors (8, 9, 35, 36), no association was found with the recurrence risk in our set of cases. This result may be explained by the inclusion of patients treated exclusively by total thyroidectomy and radioiodine therapy. This criterion was adopted to avoid treatment-related bias, which resulted in the exclusion of very low-risk cases and enriched our sample set with a more aggressive phenotype.

Our results were consistent with the association amongst *BRAF* mutation with extra-thyroidal extension and the classical histological variant (6, 35, 37). Albeit rare in our cohort, *RAS* mutation was associated with the follicular variant, as previously described (6, 38, 39). Five of 16 *RET/PTC* cases were *RET/PTC3* (*NCOA4-RET* translocation), an alteration frequently associated with ionizing radiation (40). Nonetheless, those patients have not declared any known prior exposure to radiation. Similar to previous reports, *RET/PTC* inversion was associated with lymph node involvement (39, 41). However, *RET* fusions are often observed in young patients, who have a higher frequency of lymph node metastases (39, 42). We found that 44% (7/16) of our *RET/PTC* positive cases were from patients younger than 30 years, compared to only 16% (25/158) of the *RET/PTC* negative cases. Conversely, *TERT* promoter mutation was previously described as being predominantly found in older patients (6, 43), as we observed in our dataset (range of 54–66 years). Telomerase activation is essential to cancer development by keeping the telomere length and overcoming senescence (44). Thyroid follicle cells from old individuals are *TERT*-deficient and present short-length telomeres (43). In older patients, *TERT* promoter mutation is suggested to be a consequence of the constant proliferation and activation of the telomerase, due to telomere crisis (43). Despite the small number of cases (*N* = 4) harboring both *BRAFV600E* and *TERT* promoter mutation, their clinical-pathological profile suggests a more aggressive phenotype (older patients with larger tumors and extrathyroidal extension). Even though only two patients from our whole cohort died due to the disease, one of them presented a *BRAF/TERT* concurrent mutation (diffuse sclerosing variant of PTC).

To our knowledge, no previous study has used high-throughput gene expression analysis to evaluate PTC cases with standardized treatment and long-term follow-up. Although the analysis of a homogenous cohort can eliminate the influence of some confounding factors, different histological types are frequently compared (18, 23, 45). The molecular basis of the thyroid tumor de-differentiation was studied by gene expression microarray in tumors with different degrees of aggressiveness (31 well-differentiated and 13 poorly/undifferentiated thyroid carcinomas) (23). A signature of 29 genes correctly separates 96% of tumors (42/44) according to prognosis, by grouping well-differentiated carcinomas that relapsed together with poorly/undifferentiated carcinomas (23). However, the authors included patients followed for almost 19 years in the unfavorable prognosis group and cases accompanied for <1 year categorized in the good prognosis group. A panel of 63 proteins (tissue microarray) was assessed in 12 anaplastic thyroid cancer associated with a well-differentiated component (45). The authors reported that the expression pattern of eight proteins (β-catenin, E-cadherin, thyroglobulin, topoisomerase IIα, VEGF, p53, BCL-2, and MIB-1) was able to separate anaplastic tumors from their differentiated components with 96% accuracy. Similarly, the signature of 61 differentially expressed genes found in our study was able to correctly classify PTC-RE from PTC-FCO with 87.5% accuracy (61.5% sensitivity and 97.5% specificity). No overlap between the markers found in our study with those aforementioned was found.

Among the pathways enriched by the differentially expressed genes found in our study was the FoxO signaling. This pathway is mainly activated by extracellular pro-apoptotic signals via membrane receptors, promoting downstream activation of forkhead box O3 (FOXO3), and inducing the expression of pro-apoptotic genes in the nucleus (46, 47). *BRAF*V600E directly inhibits the pro-apoptotic signals from the FoxO pathway (46). Therefore, this pathway is fundamental for the molecular pathogenesis of PTC (48). SLC2A4 (also known as GLUT4), *GADD45B*, S1PR1, and RAG1 are downstream factors induced by the FoxO pathway (49–52). These transcripts are more expressed in PTC-RE compared to PTC-FCO. However, only *GADD45B* was confirmed by RT-qPCR with the inclusion of a new group of samples (SLC2A4 and S1PR1 were also tested). Curiously, lower *GADD45B* expression was also associated with *BRAF* mutation in our dataset (by RT-qPCR) and confirmed in the TCGA dataset (RNA sequencing for *GADD45B* analysis and exome sequencing for *BRAF* genotyping) (Figure S3).

*GADD45B* is a member of the GADD45 family (Growth arrest and DNA-damage-inducible), which regulates cell proliferation through the participation of DNA replication and repair mechanisms (53), G2/M checkpoint control (54), and apoptosis (55). GADD45 family genes are rapidly induced in response to a variety of stress signals, such as ionizing radiation, pro-apoptotic inflammatory cytokines, mitogen stimulation, and xenobiotics (56). In contrast to the pro-apoptotic effect of *GADD45A* and *GADD45G* (57), *GADD45B* presents dual pro and anti-apoptotic roles (58). The mechanism responsible for inhibiting apoptosis has already been shown to attenuate JNK activation (c-Jun N-terminal kinase) (59) and induce p53 degradation (58). Decreased *GADD45B* gene expression levels have been described in several human tumors, such as lymphoma, thyroid, breast, cervical, lung, and esophageal cancers, often by epigenetic regulation (60–64). Conversely, increased *GADD45B* expression levels were associated with shorter recurrence-free and overall survival in the most prevalent and aggressive human cancer types (65). Since we have included non-neoplastic thyroid samples in the RT-qPCR analysis, it was possible to note that *GADD45B* was underexpressed exclusively in the PTC-FCO. *GADD45B* showed high expression variability in NT samples, in agreement with the TCGA dataset (Figure S3). It has been proposed that genetic and epigenetic alterations can occur in the earliest carcinogenesis steps, which can also be detected in histological “normal” tissues surrounding tumors (66, 67). *GADD45B* deficient cells have been reported to be more sensitive to ultraviolet light-induced apoptosis (68). On the other hand, increased *GADD45B* expression has been related to chemotherapy resistance (69) and survival of tumor cells resistant to ultraviolet light and gamma radiation in medium with low nutrient availability (70). Hence, it is possible that *GADD45B* deficient PTCs are more susceptible to radioiodine therapy (all patients included in our study received radioiodine therapy after surgery).

Higher expression of *GADD45B* was an independent factor for shorter disease-free survival in our internal dataset and shorter overall survival in the TCGA cohort. Likewise, high GADD45B protein expression was an independent marker of poorer prognosis in stage II colorectal cancer and a potential marker to indicate post-operative chemotherapy (71). In thyroid cancer, an accurate recurrence predictive biomarker could aid in the de-intensification of the treatment in low risk patients (72). Even though it is widely used and proven to be effective, radioiodine therapy enhances the risk for second primary tumors (mainly hematological malignancies) and alterations in salivary glands (73, 74). The evaluation of *GADD45B* expression could be incorporated in combination with clinical-pathological information in the routine to aid in the risk stratification of PTC patients. This analysis could be performed using RT-qPCR assay in post-surgical tumor samples, which has the potential to improve the indication and the intensity of radioiodine therapy and TSH suppression, and the medical surveillance frequency. Although a protein analysis using immunohistochemistry could improve even more the applicability of the test in the clinical setting, the RT-qPCR is more sensitive and has wider dynamic range quantification.

In conclusion, we showed that increased expression of *GADD45B* was an independent marker of poor prognosis of PTC, whereas genomic alterations in *BRAF, RAS, RET*, and *TERT* were not associated with the risk of recurrence in our cohort of PTC patients treated with total thyroidectomy and radioiodine therapy in a long-term follow-up.

## Data Availability Statement

All large scale data analyzed in this study are publicly available: GEO database (accession number GSE50901) and TCGA consortium (UCSC Xena Browser; https://xenabrowser.net/datapages/).

## Ethics Statement

The studies involving human participants were reviewed and approved by Ethics Committee in Human Research of A. C. Camargo Cancer Center Protocol n° 1410/10. The patients/participants provided their written informed consent to participate in this study.

## Author Contributions

MB-F, SR, and LK conceived and designed the study. MB-F and JM conducted the experiments. MB-F and FM performed bioinformatics analyses. MB-F, PD, and IS performed statistical analyses. CP performed the histopathological evaluation. JM, PD, IS, CP, and MS contributed to data interpretation. All authors participated in the preparation and approved the final version of the manuscript.

## Conflict of Interest

The authors declare that the research was conducted in the absence of any commercial or financial relationships that could be construed as a potential conflict of interest.

## Supplementary Material

Supplementary Figure 1 Flowchart summarizing the number of cases evaluated according to each methodology applied in the study. We excluded: °two samples due to poor quality of the Sanger sequencing; and ^#^four samples without conclusive results for *KRAS, HRAS* and *NRAS* mutation. ^*^Four samples were not tested for *RET/PTC* (RNA was used for expression assays); ^+^TaqMan Low Density Arrays® (TLDA); ^*a*^Taqman individual assay.

Supplementary Figure 2 Kaplan–Meier plot comparing the disease-free survival of PTC patients from TCGA according to the *GADD45B* expression (RNA sequencing). *P* values were obtained by Cox proportional-hazards regression and median expression was used as cut-off.

Supplementary Figure 3 *GADD45B* expression levels according to the *BRAF* mutation status. **(A)** Boxplots illustrating the *GADD45B* transcript evaluated by RT-qPCR in our internal sample set (NT = 15; PTC BRAF−= 61; PTC BRAF+= 118). **(B)** GADD45B transcript evaluated by RNA sequencing in the TCGA dataset (NT = 59; PTC BRAF−= 196; PTC BRAF+= 273). NT: non-neoplastic thyroid tissue; PTC: papillary thyroid carcinoma; *BRAF*^−^: Negative for BRAF mutation; *BRAF*^+^: Positive for BRAF mutation; ^****^*P* < 0.001; ^*^*P* < 0.05; NS: not significant (Tukey post-hoc test).
