# Supplementary figures and images for: GADD45B Transcript Is a Prognostic Marker in Papillary Thyroid Carcinoma Patients Treated With Total Thyroidectomy and Radioiodine Therapy
Source: Front Endocrinol (Lausanne). 2020 Apr 30;11:269. doi: 10.3389/fendo.2020.00269 (PMC7203742; doi:10.3389/fendo.2020.00269)

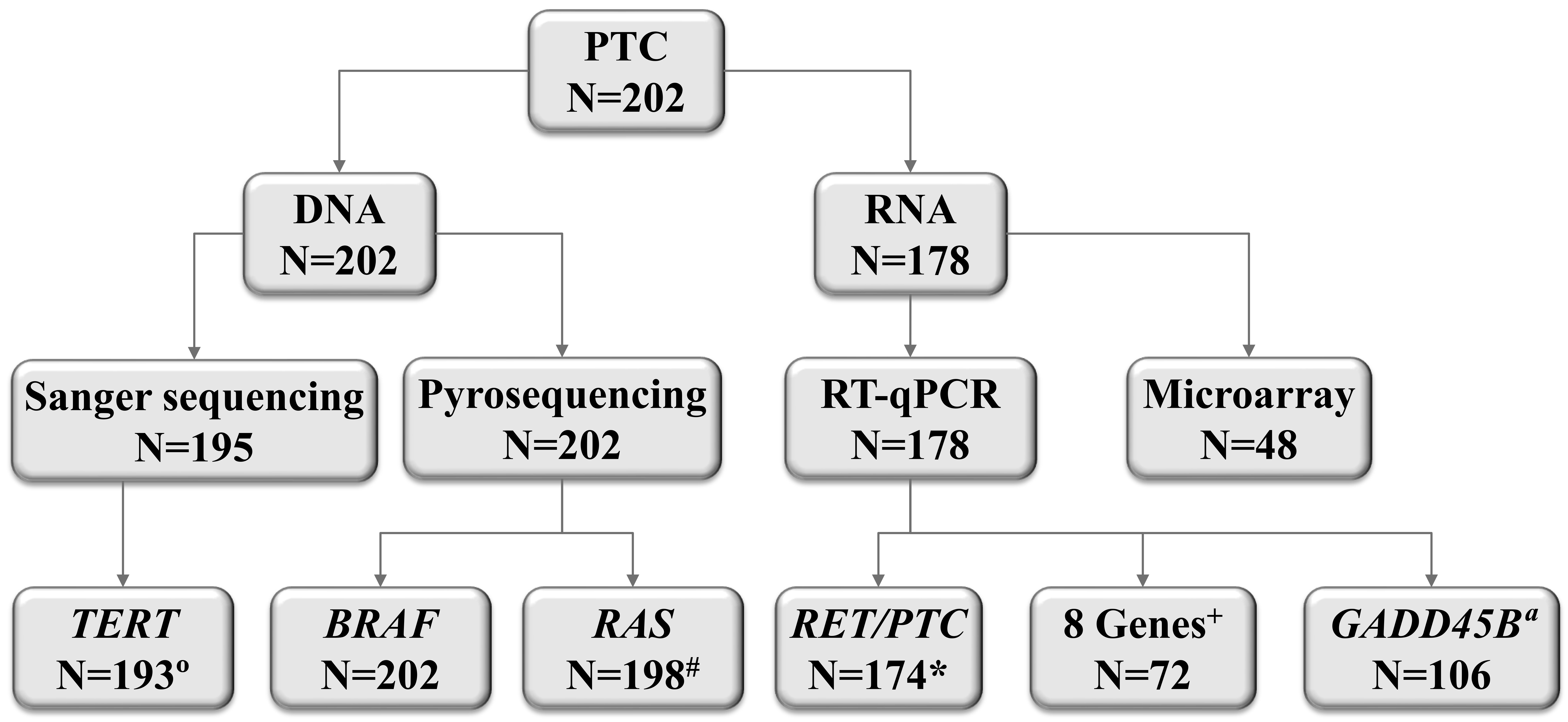

Supplement: Supplementary Figure 1 — Flowchart summarizing the number of cases evaluated according to each methodology applied in the study. We excluded: °two samples due to poor quality of the Sanger sequencing; and #four samples without conclusive results for KRAS, HRAS and NRAS mutation. *Four samples were not tested for RET/PTC (RNA was used for expression assays); +TaqMan Low Density Arrays® (TLDA); aTaqman individual assay. [file Image_1.TIF]

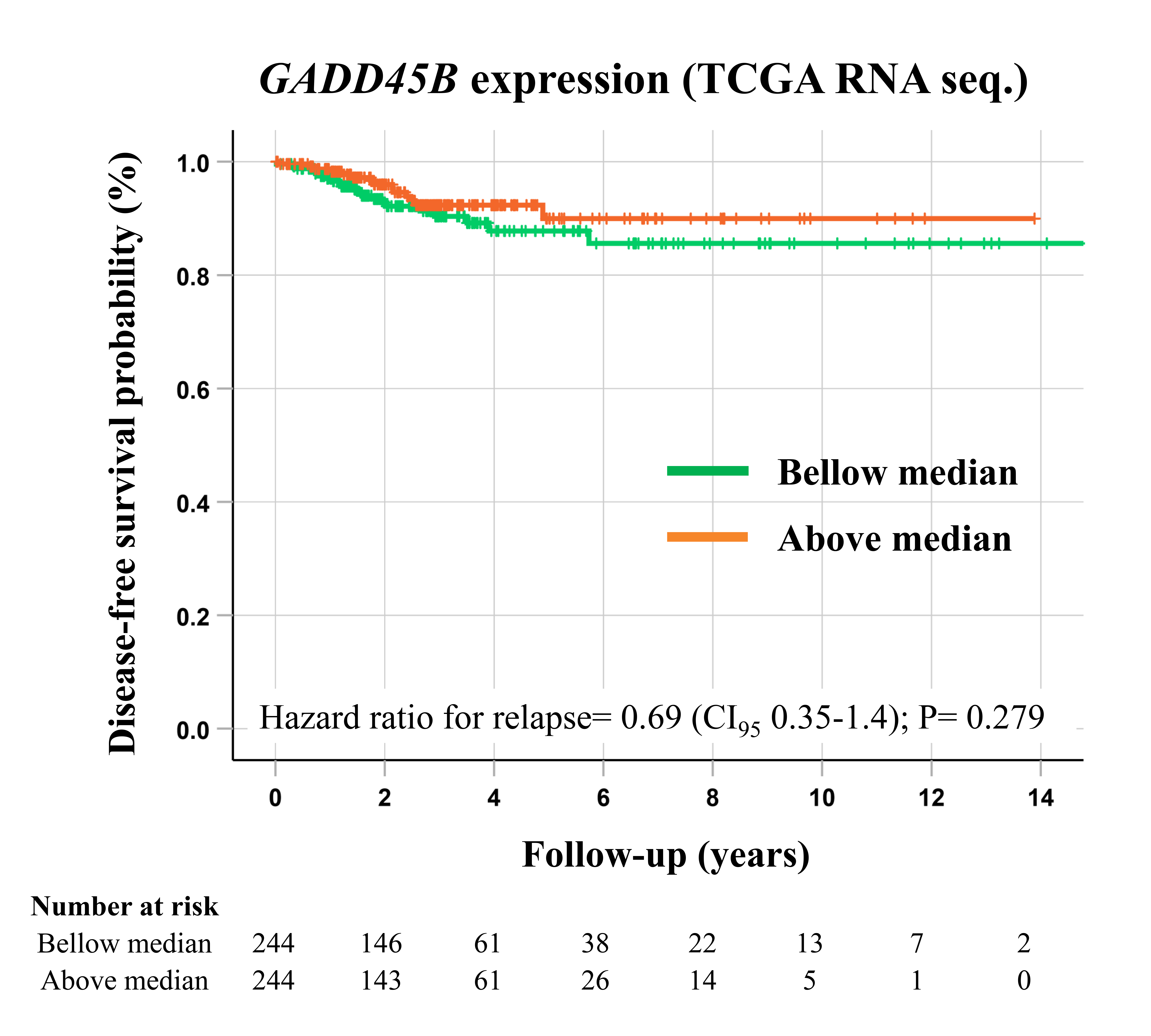

Supplement: Supplementary Figure 2 — Kaplan–Meier plot comparing the disease-free survival of PTC patients from TCGA according to the GADD45B expression (RNA sequencing). P values were obtained by Cox proportional-hazards regression and median expression was used as cut-off. [file Image_2.TIF]

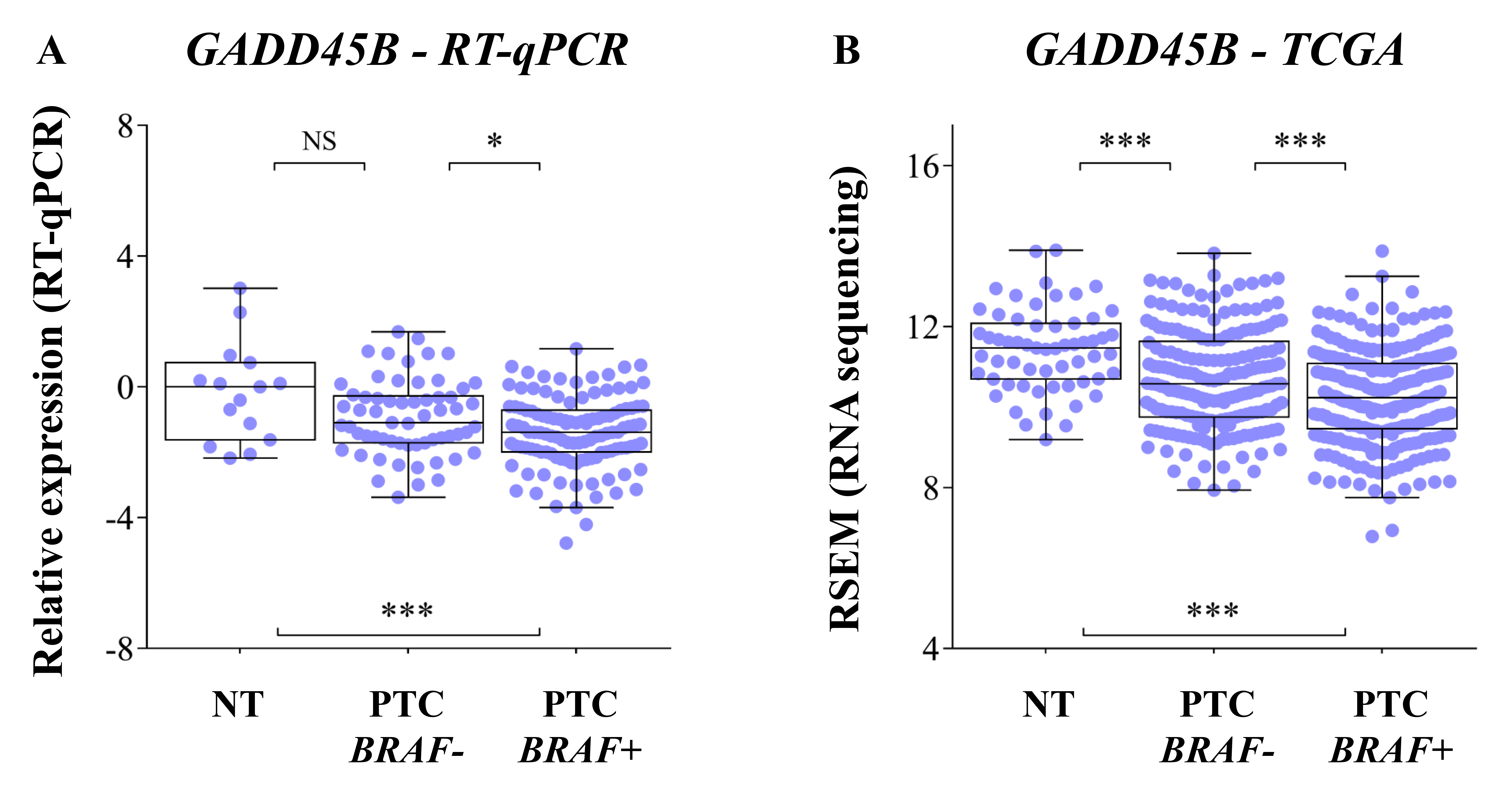

Supplement: Supplementary Figure 3 — GADD45B expression levels according to the BRAF mutation status. (A) Boxplots illustrating the GADD45B transcript evaluated by RT-qPCR in our internal sample set (NT = 15; PTC BRAF−= 61; PTC BRAF+= 118). (B) GADD45B transcript evaluated by RNA sequencing in the TCGA dataset (NT = 59; PTC BRAF−= 196; PTC BRAF+= 273). NT: non-neoplastic thyroid tissue; PTC: papillary thyroid carcinoma; BRAF−: Negative for BRAF mutation; BRAF+: Positive for BRAF mutation; ****P < 0.001; *P < 0.05; NS: not significant (Tukey post-hoc test). [file Image_3.TIF]
